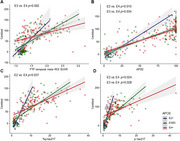# APOE Genotype Modulates the Relationship Between Plasma *p*‐tau217 and Amyloid Burden in Alzheimer's Disease

**DOI:** 10.1002/alz70856_106200

**Published:** 2026-01-10

**Authors:** Han‐Kyeol Kim, Jae Hoon Lee, Joong‐Hyun Chun, Mina Park, You Jin Kim, Tim West, Kristopher M. Kirmess, Philip B. Verghese, Daniel Connell, Joel B. Braunstein, Young Hoon Ryu, Chul Hyoung Lyoo, Hanna Cho

**Affiliations:** ^1^ Wonju Severance Christian Hospital, Yonsei University Wonju College of Medicine, Wonju, Gangwon‐do, Korea, Republic of (South); ^2^ Gangnam Severance Hospital, Yonsei University College of Medicine, Seoul, Korea, Republic of (South); ^3^ Severance Hospital, Yonsei University College of Medicine, Seoul, Korea, Republic of (South); ^4^ Gangnam Severance Hospital, Yonsei University College of Medicin, Seoul, Korea, Republic of (South); ^5^ C2N Diagnostics, LLC, Saint Louis, MO, USA; ^6^ C2N Diagnostics, LLC, St. Louis, MO, USA

## Abstract

**Background:**

This study aimed to investigate the differential association between plasma *p*‐tau217 and amyloid burden across Apolipoprotein E (APOE) genotypes.

**Methods:**

A total of 234 participants were included in the study, undergoing ^18^F‐florbetaben (FBB), ^18^F‐flortaucipir (FTP) PET, and plasma biomarker assessments, including %p‐tau217, *p*‐tau217, and APS2. Participants were categorized based on their APOE genotype: E2+ (E2/E2, E2/E3), E3/E3, and E4+ (E3/E4, E4/E4). The relationship between FBB centiloid values, FTP temporal meta‐region‐of‐interest (ROI) SUVR values, and plasma biomarkers was analyzed across these genotypes, and differences in correlations were assessed using Fisher's Z test.

**Results:**

The association between plasma *p*‐tau217 and amyloid burden varied significantly across APOE genotypes. The E2+ group exhibited the strongest correlations between FBB centiloid values and plasma biomarkers (r=0.923 for APS2, r=0.868 for %p‐tau217, r=0.826 for *p*‐tau217), which were significantly higher compared to the E4+ group (r=0.741 for APS2, *p* = 0.010; r=0.661 for %p‐tau217, *p* = 0.037; r=0.538 for *p*‐tau217, *p* = 0.024). Similarly, the E3/E3 group demonstrated stronger correlations for *p*‐tau217 (r=0.720) compared to the E4+ group (r=0.538; *p* = 0.028). Additionally, the correlation between FBB centiloid and FTP temporal meta‐ROI SUVR was significantly stronger in the E3/E3 group (r=0.698) compared to the E4+ group (r=0.413; *p* = 0.002).

**Conclusion:**

This study demonstrates that APOE genotype significantly modulates the relationship between plasma *p*‐tau217 and amyloid burden in Alzheimer's disease. Plasma *p*‐tau217 showed stronger correlations with amyloid burden in individuals with APOE E2 and E3 genotypes compared to those with the E4 genotype, highlighting its potential as a sensitive biomarker for amyloid pathology in non‐E4 carriers.